# Increasing Phosphorus Uptake Efficiency by Phosphorus-Starved Microalgae for Municipal Wastewater Post-Treatment

**DOI:** 10.3390/microorganisms9081598

**Published:** 2021-07-27

**Authors:** Aigars Lavrinovičs, Fredrika Murby, Elīna Zīverte, Linda Mežule, Tālis Juhna

**Affiliations:** 1 Water Research and Environmental Biotechnology Laboratory, Faculty of Civil Engineering, Riga Technical University, P. Valdena 1, LV-1048 Riga, Latvia; elina.ziverte_1@rtu.lv (E.Z.); linda.mezule@rtu.lv (L.M.); talis.juhna@rtu.lv (T.J.); 2 Institute for Environmental Solutions, “Lidlauks”, Priekuļi Parish, LV-4126 Priekuļi County, Latvia; 3 Department of Civil, Environmental and Natural Resources Engineering, Luleå University of Technology, 97187 Luleå, Sweden; fermur-5@student.ltu.se

**Keywords:** microalgae, phosphorus starvation, municipal wastewater, nutrient removal, polyphosphate, alkaline phosphatase activity

## Abstract

Four microalgal species, *Chlorella vulgaris*, *Botryococcus braunii*, *Ankistrodesmus falcatus*, and *Tetradesmus obliquus* were studied for enhanced phosphorus removal from municipal wastewater after their exposure to phosphorus starvation. Microalgae were exposed to phosphorus starvation conditions for three and five days and then used in a batch experiment to purify an effluent from a small WWTP. After 3-day P-starvation, *C. vulgaris* biomass growth rate increased by 50% and its PO_4_ removal rate reached > 99% within 7 days. *B. braunii* maintained good biomass growth rate and nutrient removal regardless of the P-starvation. All species showed 2–5 times higher alkaline phosphatase activity increase for P-starved biomass than at the reference conditions, responding to the decline of PO_4_ concentration in wastewater and biomass poly-P content. The overall efficiency of biomass P-starvation on enhanced phosphorus uptake was found to be dependent on the species, N/P molar ratio in the wastewater, as well as the biomass P content.

## 1. Introduction

Globally, phosphorus (P) is a major nutrient causing eutrophication of aquatic ecosystems. Among the many phosphorus sources, effluents from wastewater treatment plants (WWTPs) provide significant loading of P into the surface waters. Recovery of P from wastewaters is lately gaining more attention [1]. Chemical precipitation and enhanced biological (bacterial) uptake are the main methods for additional P removal used in WWTPs. Although these methods are well established and applied in the large WWTPs, their use in small WWTPs is often not practiced due to legislative acts. For instance, in the European Union the regulation on municipal wastewater treatment (Directive 91/271/EEC) does not state any limits for permissible P concentration in the effluent from WWTPs operating in small agglomerations with less than 2000 p.e. [2]. Furthermore, these methods still cannot reduce the P concentration to ultra-low level (<0.1 mg P L-1). Scaling the alternative P removal methods to small WWTPs results in high capital and operational costs and overall system complexity [3], and is rather detrimental as the obtained phosphorus reduction rates in small scale are often insufficient and fail to meet environmental safety standards. 

A promising alternative to the traditional wastewater P removal and recovery methods is the use of microalgae systems [4,5]. Many studies, mostly in lab scale, have shown that various microalgal strains or mixed cultures are good candidates for wastewater post-treatment as they can reduce the phosphorus concentrations in wastewaters to ultra-low levels [6]. Still, there are often reports on incomplete P removal rates over longer time periods [7,8,9] which can be viewed as a drawback for efficient microalgae-based wastewater post-treatment step. On the other hand, certain conditions are known at which algal cells can consume more phosphorus than their production requires. Enhanced algal phosphorus uptake (EAPU) and storage of excess phosphorus in algal cells can be achieved by manipulation with external phosphorus availability [10,11]. The two approaches known to cause this phenomenon are the luxury P uptake mechanism [12,13] and phosphorus starvation [14], which are initiated by excess phosphorus availability and phosphorus limited conditions, respectively. Both biomass manipulation approaches can result in ten times higher phosphorus uptake rate [15] when compared to regular biomass phosphorus consumption. In addition to more efficient phosphorus uptake, biomass exposure to nutrient stress is known to promote valuable substance production by algal cells [16], thus adding value to the produced biomass and providing an opportunity for financial return.

The existing knowledge on enhanced phosphorus uptake induced by algal biomass starvation shows it as a promising feature for algae-based wastewater post-treatment technologies. Though, several ambiguities still need to be specified before proceeding with EAPU implementation into full scale algae-based wastewater post-treatment systems: (1) it is unclear whether biomass manipulation for EAPU is limited to specific strains; (2) more information is needed for indicators that characterize the status of biomass phosphorus deficiency, and (3) it should be clarified under which biochemical conditions the EAPU is more efficient, and what are the physical and biochemical conditions that hampers this process. 

This paper investigates the impact of various P-starvation periods on phosphate removal efficiency from municipal wastewater. In the batch experiments we compared the performance of different microalgal species in terms of their biomass growth and nutrient (phosphate and nitrate) removal rates to estimate their applicability for wastewater treatment. Biochemical parameters such as polyphosphate accumulation and alkaline phosphatase activity were assessed for quantitative characterization of algal biomass P-starvation.

## 2. Materials and Methods

### 2.1. Microalgae Strain Selection and Culturing Conditions

The photoautotrophic microalgae strains *Tetradesmus obliquus* (CCAP 276/10), *Botrycoccus braunii* (CCAP 807/1), *Chlorella vulgaris* (CCAP 211/11B), and *Ankistrodesmus falcatus* (CCAP 202/5C) were used in this study. These strains were selected for their reported ability to grow in various types of wastewaters, reduce nutrient content, and produce valuable substances such as carbohydrates and lipids.

Algae cells were pre-cultured in a sterile BG-11 growth medium [17]. Every two weeks half of the culture volume was replaced by freshly prepared growth medium. The cell culturing was done in a 1000 mL Pyrex^®^ bottles with constant 10 L h^−1^ aeration. Tubular (T5) fluorescent lamps with the blue-red spectrum were used as a light source. The provided photosynthetically active radiation was 180 μmol m^2^ s^−1^ at a 16:8-h lighting regime. The ambient temperature was between 25 and 27 °C and the pH ~7.5. 

For the phosphorus starvation, algal biomass was prepared by washing it with demineralized water. At first it was centrifuged (4000× *g*, 2 min) and the growth medium supernatant was replaced by demineralized water. Afterwards the biomass was vortexed and centrifuged again. After the centrifugation, the supernatant was discarded, and biomass was inoculated in a phosphate-free (without K_2_HPO_4_) BG-11 growth medium. Microalgae that have been exposed to P-deficit conditions for 3 and 5 days were used for the experiments and biomass produced with sufficient phosphate availability was used as a reference.

### 2.2. Wastewater Source

Wastewater from a treatment plant (WWTP) in Roja (Latvia) village (57.506465 N, 22.809536 E) was used for the research. The plant provides service to two villages (Roja and Rude) with a total population of ~3000 inhabitants. It also accepts sewage from three local fish processing factories that accounts for 21.4% of the total 30,846 m^−3^ of sewage the plant received in 2019. 

Secondary wastewater after biochemical oxidation and secondary settling was double filtered through membrane filters with pore sizes of 0.45 and 0.2 μm to remove bacterial pollution and microparticles prior all experiments. The chemical content of the wastewater after the filtration is shown in Table 1.

### 2.3. Experimental Setup 

The experiments were conducted in batch regime, in 1000 mL Pyrex^®^ bottles with working volume of 800 mL, at room temperature (25–27 °C) for 10 days. The algae-wastewater suspensions were mixed by aeration (10 L h^−1^) and supplemented with 1% (*v*/*v*) CO_2_. The bottles were illuminated with fluorescent light with blue-red spectrum with the intensity of 180 μmol m^2^ s^−1^ at a 16:8-h lighting regime. Sampling was performed daily, each time taking a 70 mL sample to detect the biomass change, nutrient concentration, the amount of cellular polyphosphate, alkaline phosphatase activity, pH, and temperature.

### 2.4. Analytical Procedures

#### 2.4.1. Nutrient Analysis

To follow the algal nutrient consumption from wastewater, concentration changes for orthophosphate, nitrate as well as total nitrogen and total phosphorus were observed. Measurements were done using a spectrophotometer (HACH LANGE DR 3900, Loveland, CO, USA) and commercial reagent sets for each parameter analysis. Total nitrogen and nitrate concentrations were measured using alkaline persulfate digestion method [18] and chromotropic acid method [19], respectively. Total phosphorus and phosphate concentrations were measured using the ascorbic acid method [20]. The percentage nutrient removal was obtained using Equation (1):

(1) $$R_{N,P}=\frac{\left(C_{0}-C_{i}\right)}{C_{0}}\times 100$$

where *R_N,P_* is the removal (%) of nitrogen and phosphorus, *C_0_* is the initial nutrient concentration, and *C_i_* is the nutrient concertation at the end (or specific day) of experiment.

#### 2.4.2. Determination of Biomass Concentration Change

Algae biomass growth was detected spectrophotometrically using an UV-Visible spectrophotometer (Thermo Scientific Genesys 150, Waltham, MA, USA). Light absorbance was measured at 680 nm which is proportional to the change of cell number in most unicellular organisms [21]. The OD_680_ values were kept bellow 1.2. and the samples were diluted appropriately when exceeding this value. The dry weight of algal biomass was used for making growth curves. The relationship between algal dry weigh biomass (g L^−1^) and light absorbance value at OD_680_ was described by the individual equations constructed for each algal species (Table 2).

The specific biomass growth in the exponential phase was estimated by Equation (2):

(2) $$\mu \left({\mathrm{day}}^{-1}\right)=\frac{\ln\left(N_{2}/N_{1}\right)}{\left(t_{2}-t_{1}\right)}$$

where *N_1_* and *N_2_* are the measured dry biomass at time *t_1_* and *t_2_*, respectively. The biomass productivity (Pr) was calculated according to the Equation (3):

(3) $$\mathrm{Pr}=\frac{\left(DW_{i}-DW_{0}\right)}{\left(t_{i}-t_{0}\right)}$$

where *DW_i_* and *DW_0_* are dry biomass (g L^−1^) at time *t_i_* and *t_0_* (initial time), respectively.

#### 2.4.3. Polyphosphate Detection and Quantification

Polyphosphate content in algal biomass was measured following the protocol of Mukherjee and Ray [22]. Briefly, the polyphosphates were extracted from microalgal biomass through a series of physical and chemical procedures. Suspension samples were concentrated to harvest enough biomass (>0.05 g fresh weight) for the extraction process. At first the algal cells were disrupted using an ultrasonic processor (Cole-Palmer Instruments, 130-watt, Vernon Hills, IL, USA), treating the sample for five minutes at 30 kHz. Afterwards the disrupted cell samples were heated at 100 °C for 2 h. Then a mixture of chloroform and isoamyl alcohol (24:1) was added to the biomass and mixed vigorously. The suspension was centrifuged at 13,520 g for 15 min. Afterwards the supernatant was collected and mixed with 0.2 N acetic acid and toluidine blue solution (stock conc. 30 mg L^−1^) for spectrophotometric light adsorption measurement at 630 nm. Biomass polyphosphate concentration (μg mg^−1^) was calculated against a calibration curve constructed using a sodium phosphate glass Type-45 (Sigma-Aldrich, St. Louis, MO, USA) as a polyphosphate standard. 

#### 2.4.4. Alkaline Phosphatase Activity Detection

The alkaline phosphatase (AP) activity was estimated by using a method which involves para-nitrophenylphosphate disodium hexahydrate (p-NPP, Sigma-Aldrich, St. Louis, MO, USA) hydrolysis to para-nitrophenol (p-NP). Total of 3 mL sample was mixed with 1 mL Tris HCl buffer (pH 9.5) and 0.4 mL p-NPP (0.5 mg mL^−1^). The mixture was incubated at 37 °C for 1 h in the dark. The yielded p-NP was measured spectrophotometrically at 405 nm. p-NP amount was calculated against a calibration curve that was constructed using a p-NP as a standard. The result was used as an indicator for AP activity. A control containing no biomass was included in the routine, and its OD_405_ reading was subtracted from the hydrolyzed value. AP activity was expressed as p-NP flux from dry-weight biomass per hour.

### 2.5. Determination of Nutrient Uptake Kinetics 

Nutrient uptake rates and biomass nutrient consumption for NO_3_-N and PO_4_-P were estimated for the biomass exponential growth rate period, from experiment day 1–5. The biomass nutrient consumption *V_N(P)_* (mg *N(P)* g^−1^ DW) was calculated as nutrient concentration change over biomass concentration increase:

(4) $$V_{N\left(P\right)}=\frac{cN{\left(P\right)}_{0}-cN{\left(P\right)}_{i}}{b_{i}-b_{0}}$$

where *cN(P)*_0_ is the initial nutrient concentration, *cN(P)_i_* is the nutrient concentration at a specific time, *b*_0_ is the initial biomass concentration, *b_i_* is the biomass concentration at a specific time. 

The nutrient uptake rate *k* (d^−1^) was calculated as biomass nutrient consumption over biomass growth rate:

(5) $$k=\frac{V_{N\left(P\right)}}{\mu }$$

where *V_N(P)_* is the biomass nutrient consumption from Equation (4) and *μ* is the biomass growth rate from the Equation (2).

### 2.6. Data Analysis

Parametric one-way ANOVA test was used to detect the significant differences among different biomass P-starvation periods on algal biomass growth, nutrient consumption, biomass polyphosphate accumulation, and APA for each algal specie. Tukey post-hoc test was used to detect pairwise differences between the treatments. The limit of statistical significance in all tests was set to α ≤ 0.05. Results are presented as mean values (*n* = 3). Statistical analyses were conducted using IBM SPSS Statistics version 23 software (Armonk, NY, USA). The obtained results were visualized using R: A Language and Environment for Statistical Computing, version 4.0.5 (Vienna, Austria). 

## 3. Results and Discussion

### 3.1. Algal Growth and Biomass Production

**Figure 1:**
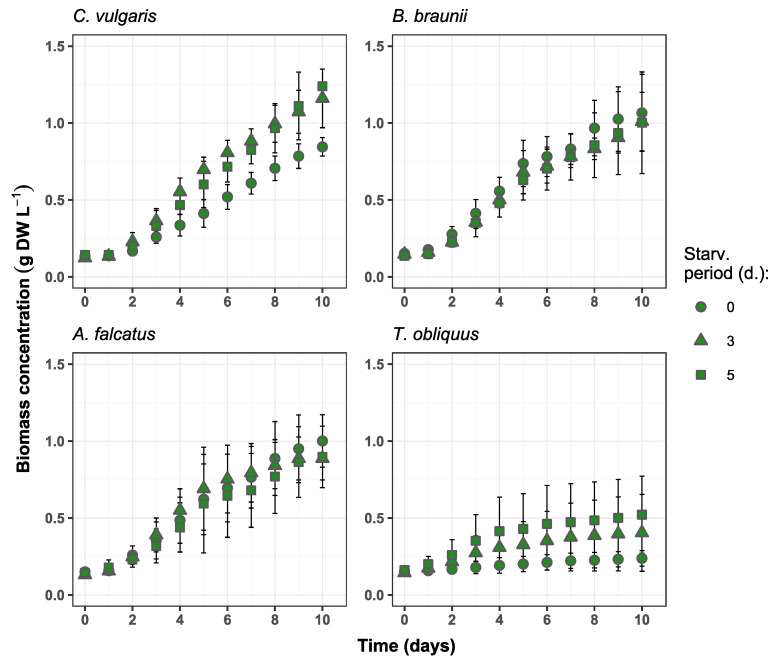
Biomass concentration changes over time in the experiment batches for *C. vulgaris*, *B. braunii*, *A. falcatus* and *T. obliquus* (means ± SD, *n* = 3).

**Figure 2:**
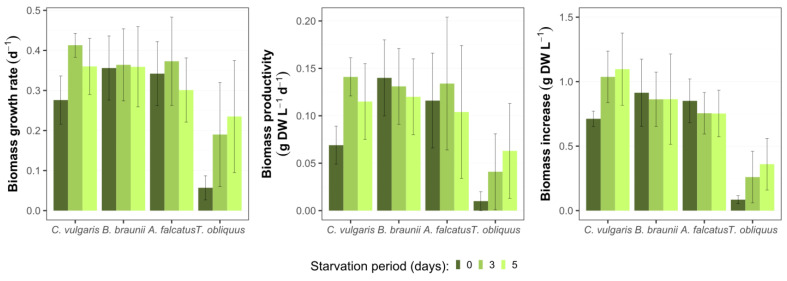
Biomass growth parameters for *C. vulgaris*, *B. braunii*, *A. falcatus*, and *T. obliquus* in batch conditions after various P-starvation periods within ten days (means ± SD, *n* = 3).

All species used for the experiment could produce biomass in secondary wastewater. No negative effect (e.g., growth inhibition or cell death) on the biomass growth were observed due to biomass exposure to phosphorus deficiency (Figure 1). Differences in biomass growth rate (*μ*), biomass productivity (Pr), and biomass increase were observed among the species and applied P-starvation periods (Figure 2). *C. vulgaris* showed higher *μ* after its biomass exposure to P-deficiency. Comparing to the reference conditions, the biomass growth rate after 3- and 5-day starvation was 49.7 and 30.3% higher, respectively. Moreover, the productivity of *C. vulgaris* reached 104.9% after 3-day starvation. Unlike other species, after 3- and 5-day P-starvation *C. vulgaris* showed 45.5 and 55.0% higher biomass increase respectively, than in the reference batch. The concentration change of P-starved biomass showed no statistically significant difference (*p* > 0.05) from the reference biomass.

The observed enhanced biomass growth of *C. vulgaris* as a response to P-starvation is partly in line with the observations by Hernadez et al. [14], who obtained a significant biomass growth increase for *C. vulgaris* in domestic wastewater after P-starvation for 3 days. Moreover, rapid biomass growth of *C. vulgaris* after 3-day P-starvation was demonstrated by Solovchenko et al. [23]. Their given explanation behind such an outcome is a rapid P*i* uptake by starved biomass after phosphorus re-feeding, further synthesis of phosphorus to polyphosphate that is a source of energy and metabolic processes for cells, and its intensive consumption for new cell production. 

For *B. braunii* biomass concentration change at both P-starvation periods did not show significant difference (*p* > 0.05) from the reference batch. The biomass growth rate and productivity were 6.0 and 10.0% higher, respectively, for both starvation periods. The observed biomass increases for 3- and 5-day starved biomass was 3.3 and 7.3% lower than in the reference batch. Other studies show inconsistent results for *B. braunii* biomass production in wastewater. For instance, Aravantinou et al. [24] demonstrated more than 10 times lower biomass growth rate of *B. braunii*, than obtained in this study. Álvarez-Díaz et al. [25] argued that *B. braunii* is unsuitable for production in municipal wastewater due to its low growth rate and productivity. On the other hand, Ruangsomboon [26] showed that *B. braunii* biomass production increased with higher phosphate concentration, and the rates were comparable to the present study. This specie has also shown good productivity in industrial wastewaters with high carbon content [27,28]. Thus, different studies suggest that *B. braunii* is a nutrient demanding specie and phosphorus stress is rather adverse for its biomass production. On the other hand, the results from present study show that the selected *B. braunii* strain can tolerate phosphate stress and grows well in wastewater after exposure to different P-starvation periods.

The growth rate of *A. falcatus* biomass increased by 8.8% after 3-day P-starvation but was 12.0% lower than the reference after 5-day P-starvation. The biomass productivity of *A. falcatus* was 12.8% higher for 3-day starved and 15% lower for 5-day starved, while the biomass increase was 11.3% lower for both starvation periods than in the reference batch. No significant difference (*p* > 0.05) was observed between the growth of P-starved and reference biomasses. Compared to other species *A. falcatus* showed less productive performance for all biomass growth parameters. A study by Álvarez-Díaz et al. [25] showed, that in terms of biomass growth rate and productivity *A. falcatus* was outperformed by *C. vulgaris* and *B. braunii*. Compared to the reference conditions, *A. falcatus* reached slightly higher biomass growth rate and productivity after 3 days of P-starvation. A possible reason for such a behavior by *A. falcatus* is its high lipid productivity, which is boosted under P-deficiency conditions. Being a key substance for algal cell metabolism, excessively accumulated lipids can enhance cell-doubling process. A similar outcome was observed by Álvarez-Díaz et al. [29] where *A. falcatus* showed increased growth rate under P-deficiency and produced more lipids than under regular conditions. On the other hand, a large standard deviation was obtained for *A. falcatus* growth rate and productivity, so the present result is rather ambiguous and cannot be genuinely related to phosphorus stress conditions.

*T. obliquus* biomass growth showed considerable improvement after P-starvation. Its biomass growth rate increased by 232 and 310% after 3- and 5-day P-starvation, respectively, comparing to the reference conditions. The biomass productivity was 316 and 541% higher and biomass increase was 205 and 323% after 3- and 5-day P-starvation compared to the reference conditions. However, it remains unclear what caused such an improvement in biomass growth. At all growth parameters *T. obliquus* showed markedly worse performance than the other species. Biomass concentration change of *T. obliquus* after 3- and 5-day starvation significantly differed (*p* < 0.05) from the reference conditions. However, the biomass growth values for *T. obliquus* show large uncertainty as the standard deviation is more than ±50% from the mean biomass growth value for all treatments. 

The observed biomass growth rates, productivity, and increase as well as the response to phosphorus deficiency emphasize *C. vulgaris* and *B. braunii* as more appropriate for nutrient removal at wastewater post-treatment than the other studied species. The ability of *C. vulgaris* to grow in various types of wastewaters have been widely reported [30] and it is often selected for commercial purposes [31]. *B. braunii* is known as a carbohydrate producer and has a potential for bioenergy production [32]. If high biomass productivity can be achieved, *B. braunii* is a suitable candidate for resource recovery from wastewater. Both *A. falcatus* and *T. obliquus* are often viewed as a potential species for high economic return due to their lipid productivity [33,34]. However, lower growth rate and productivity obtained in the present study deems them less appropriate for wastewater post-treatment with possible economical return.

### 3.2. Nutrient Removal 

**Figure 3:**
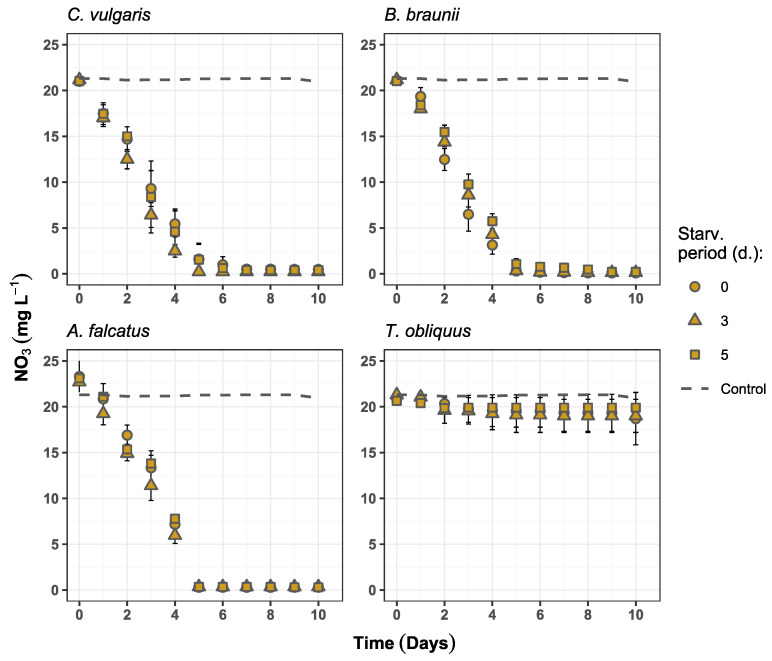
Nitrate concentration changes over time in the experiment batches for *C. vulgaris*, *B. braunii*, *A. falcatus*, and *T. obliquus* (means ± SD, *n* = 3).

All species, except *T. obliquus*, showed high nutrient removal rates, reaching more than 97 and 91% reduction of nitrate and phosphate, respectively (Table 3). Nitrate removal was not affected by prior biomass exposure to P-deficiency conditions and showed no statistically significant difference (*p* > 0.05) among the treatments for any of the species. The maximum nitrate removal for *C. vulgaris*, *B. braunii*, and *A. falcatus* was obtained on days 5–6 (Figure 3). 

**Figure 4:**
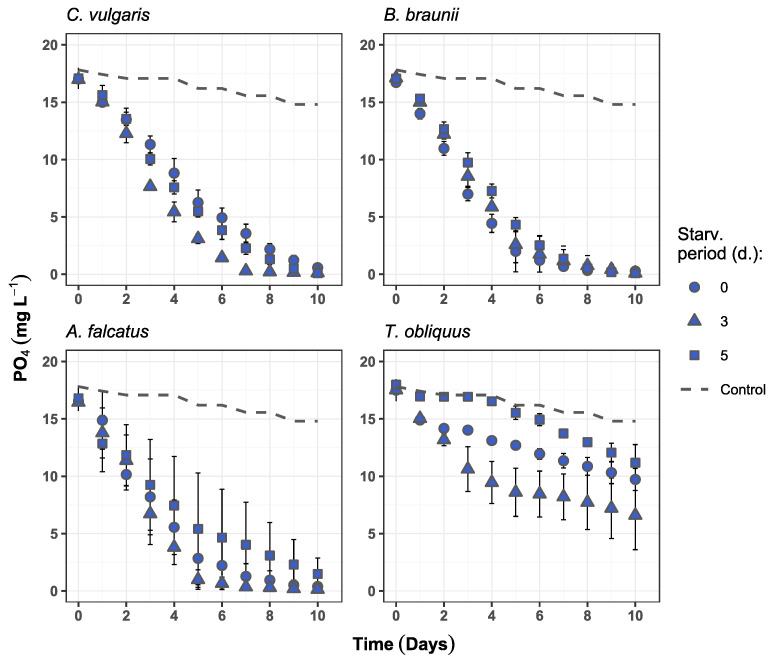
Phosphate concentration changes over time in the experiment batches for *C. vulgaris*, *B. braunii*, *A. falcatus*, and *T. obliquus* (means ± SD, *n* = 3).

Higher phosphate removal rates were observed for *C. vulgaris* and *B. braunii* after prior biomass exposure to P-deficiency and reached near-complete (>99%) PO_4_ reduction. *A. falcatus* at the same time showed near-complete PO_4_ reduction only after 3-day phosphorus starvation. In general, the highest phosphate removal rate was recorded after 10 days of growth, except for 3-day starved *C. vulgaris* and *A. falcatus* which both reduced phosphate by 99.2% on day 7 (Figure 4). *T. obliquus* could reduce PO_4_ content by 62.4% after 3-day P-starvation at the end of the experiment. This treatment showed significantly higher (*p* < 0.05) PO_4_ reduction than the 5-day starved biomass. All other species showed no statistically significant differences (*p* > 0.05) among the treatments in PO_4_ removal. 

**Figure 5:**
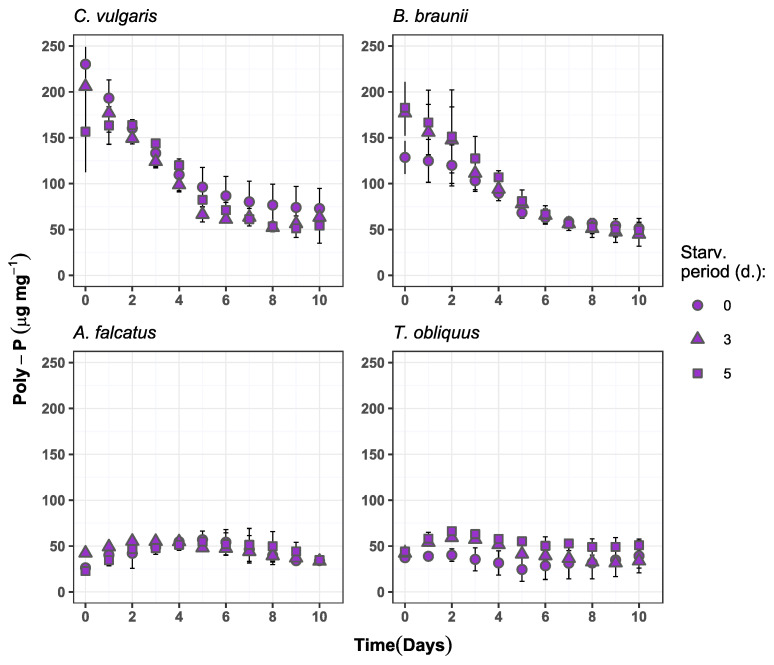
Biomass polyphosphate content change over time in the experiment batches for *C. vulgaris*, *B. braunii*, *A. falcatus* and *T. obliquus* (means ± SD, *n* = 3).

Although slight increase in phosphate removal for certain species was observed after biomass P-starvation, it can be viewed as underperformance. Similar studies, where biomass P-starvation is performed to enhance the phosphate uptake, show near complete phosphate reduction in the range from within few hours to two days [10,23]. The obtained results highlight two major bottlenecks behind slow phosphate uptake by P-starved biomass. First, the low N/P ratio in the wastewater as well as faster removal of nitrogen indicate the importance of nitrogen limitation. This results in the preference for nitrate over phosphate, omitting the possible biomass status of P-deficiency. Second, rapid phosphate removal by P-starved biomass in other studies was achieved at conditions where cellular phosphorus was depleted. However, in this study to mimic practical conditions, internal biomass phosphorus reserve in the form of polyphosphate was still available after biomass exposure to P-deficiency (Figure 5). This condition emphasizes the prospective complexity of P-starvation implementation in pilot-scale wastewater post-treatment—complete biomass polyphosphate depletion requires long exposure to P-deficiency.

Evidently, the phosphate concentration decreases slightly faster in batches with 3-day P-starved *C. vulgaris* and *A. falcatus* biomass during its exponential growth period (Figure 3). However, when assessing the phosphate removal efficiency in terms of PO_4_ reduction per daily produced biomass, the P-starvation effect on PO_4_ uptake becomes less distinct. The calculated biomass nutrient consumption (V) and nutrient uptake rates (k) show that *C. vulgaris* and *A. falcatus* removed phosphate more efficiently without prior biomass P-starvation (Table 3). Both V and k represent nutrient reduction at biomass exponential growth phase, which was observed from day 1 to 5. *C. vulgaris* biomass consumed at least 30% more phosphate than the previously starved biomass. The biomass phosphate uptake rate was 45–55% higher by *C. vulgaris* and 15–22% higher for *A. falcatus* for the reference biomass. Although it was observed that the biomass production of P-starved cells increased, the produced biomass to P-uptake ratio was lower than at the reference conditions, indicating biomass growth inhibition. Such a condition can be observed after biomass P-deficiency [35] and is associated to cell respiratory metabolism repression [36].

In terms of phosphate and nitrate removal, all species used in this study have their benefits. *C. vulgaris* and *A. falcatus* show indications of enhanced phosphate uptake after P-starvation. Thus, they seem more appropriate for application in pilot-scale systems for wastewater post-treatment. However, the N/P ratio must be adjusted and kept at optimum (around 16/1) to achieve rapid phosphate removal. *B. braunii* seemingly retains its removal efficiency after exposure to P-deficiency, but it is unknown whether P-starved biomass inoculated in the medium with optimum N/P ratio would improve the biomass phosphate consumption and uptake rate.

### 3.3. Biomass Poly-P Content and AP Activity as Indicators for P-Deficiency 

It is a generally accepted knowledge that microalgae primarily assimilate the inorganic form of phosphorus for their growth [37,38]. Therefore, low availability or complete lack of inorganic phosphorus from external sources is viewed as the major driver of algal P-deficiency. On the other hand, microalgae can temporarily survive from its internal P reserves. The internal P is extensively acquired after temporary P deprivation and is stored within the cell as polyphosphate granules [39]. Another alternative phosphorus source is the dissolved organic phosphorus, that can be enzymatically hydrolyzed to the bioavailable inorganic P [40]. Thus, fluctuations in cellular poly-P content and high extracellular alkaline phosphatase (AP) activity indicate on P-deficiency and can be applied to monitor P-starvation.

The obtained results on the biomass polyphosphate content show inconsistency among the species (Figure 5). *A. falcatus* and *T. obliquus* showed increase in biomass poly-P content during the first half of the experiment period. For *A. falcatus* the 3- and 5-day starved biomass increased its poly-P content by 23 and 58%, respectively, compared to its initial content. For *T. obliquus* the poly-P content during experiment increased by 28 and 33% for 3- and 5-day starved biomass, respectively, and was accumulating 5–6 times more poly-P than the reference biomass. However, this observed increase of poly-P after P-starvation is many orders of magnitude lower than reported in other studies [10,23,41]. The initial poly-P content for *C. vulgaris* biomass was 230, 205, and 156 μg mg^−1^ for the reference, 3- and 5-day starved biomass, respectively. Still, the poly-P content showed a declining dynamic indicating to a constant consumption of internal P reserves. Only 5-day starved *C. vulgaris* biomass showed 4.4% accumulation during first two days, followed by poly-P content reduction until the end of the experiment period. Similar biomass poly-P content change was observed for *B. braunii* at all treatments—high initial biomass poly-P content and its decrease during the experiment period. Among all species, only *T. obliquus* showed significantly higher (*p* < 0.05) poly-P accumulation by P-starved biomass compared to the reference conditions.

The observed initial biomass poly-P concentration and its changes over the experiment period suggest that neither of the species developed a clear P-deficiency condition during the biomass P-starvation that might resemble full-scale situation. Thus, the selected P-starvation periods were too short to reduce the poly-P content to a level where P-deficiency is induced and subsequently enhances phosphate uptake and polyphosphate storage. Such an outcome is not in line with reports from other studies, where 3–5 days of P-starvation have entirely depleted the cellular poly-P content [23,41]. It has been previously demonstrated that the cellular poly-P accumulation and consumption rate can be affected by the biomass growth phase [42]. Accordingly, the result from this study indicates that before the P-starvation *C. vulgaris* and *B. braunii* were at an early stationary growth phase when poly-P was accumulated to overcome extended periods without external orthophosphate availability. Contrary, *A. falcatus* and *T. obliquus* were at the exponential growth phase, when they actively used the acquired internal P reserves for biomass production. 

The results on biomass poly-P content dynamics suggests that it is not directly related to external P availability. It also depends on the cell growth phase as well as light intensity, temperature, and pH [12,42]. Therefore, the biomass poly-P content alone is not a reliable indicator for biomass P-deficiency. 

The observed alkaline phosphatase (AP) activity partly supports the incomplete P-deficiency status for all species. The P-starved biomass for all four species showed lower AP activity than the reference biomass. Concurrently, the P-starved biomass showed more rapid increase in AP activity relative to its initial value. For all species, the AP activity dynamics for P-starved biomass significantly differed (*p* < 0.05) from the reference biomass.

**Figure 6:**
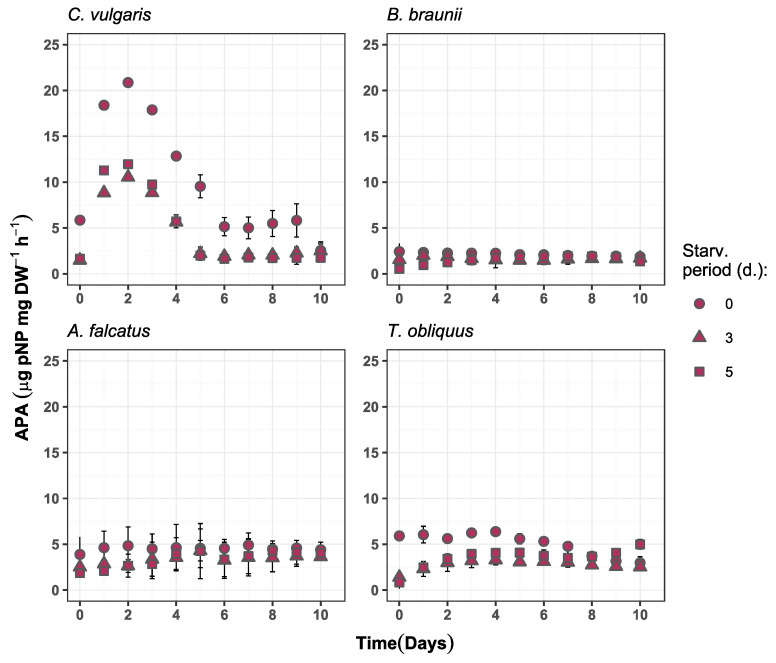
Alkaline phosphatase activity change over time in the experiment batches for *C. vulgaris*, *B. braunii*, *A. falcatus*, and *T. obliquus* (means ± SD, *n* = 3).

Differences in AP activity change during the experiment period was observed among the species. For *C. vulgaris* the AP activity developed differently from other species, showing increase during the first two days, followed by decline and a steadily low value until the end of the experiment (Figure 6). For *B. braunii*, *A. falcatus*, and *T. obliquus* an increase in AP activity was observed for the P-starved biomass while for the reference biomass it mostly decreased or showed no change.

**Figure 7:**
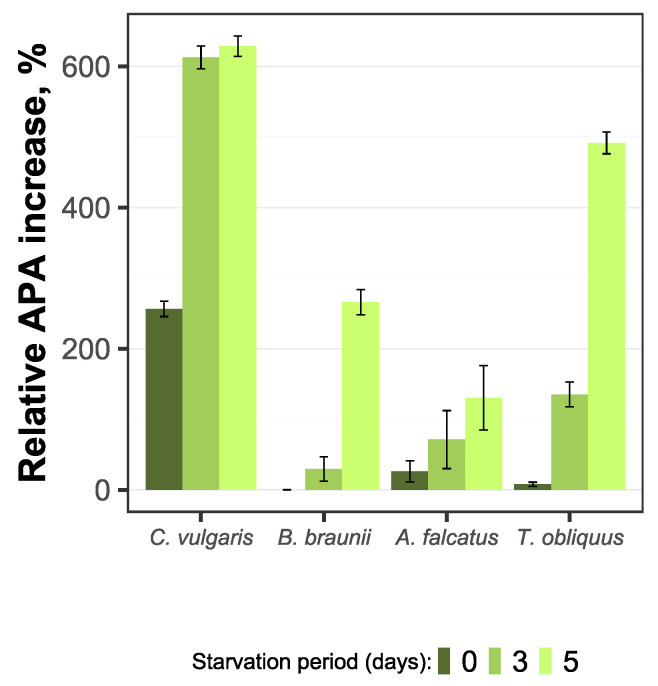
Relative increase of alkaline phosphatase activity for *C. vulgaris*, *B. braunii*, *A. falcatus*, and *T. obliquus* (means ± SD, *n* = 3).

The initially low AP activity for P-starved biomass indicate on phosphorus availability from internal reserves during the exposure to P-starvation. Under such condition there was no need for enhanced release of enzymes to hydrolyze organic P to a bioavailable inorganic form. On the other hand, for all species the AP activity showed increase by P-starved biomass as the available phosphorus resource was decreasing. Moreover, a multifold increase of AP activity during the biomass exponential growth phase relative to its initial value was observed for the P-starved biomass by all species (Figure 7). This indicates that P-starved algal cells are potentially more sensitive to bioavailable phosphorus decrease and releases the phosphatase to compensate P-deficiency.

AP activity is commonly used as an indicator for algal P-deficiency in natural aquatic ecosystems [43,44,45]. However, this study shows that AP activity also detects P-deficiency in laboratory algal species grown in real wastewater. Thus, AP activity has potential application in engineered systems for algae-based wastewater treatment supplemented with biomass P-starvation for enhanced phosphorus uptake. The monitoring of AP activity gives more control over the manipulation with algal P-deficiency in wastewater post-treatment phase. It would allow a prompt detection of P-deficiency status development and optimize the overall hydraulic and biochemical performance of phosphorus removal. This way high phosphorus removal rates can be achieved in short time periods, and ultimately increase the efficiency of small WWTPs performance.

## 4. Conclusions

Performance of four different microalgal species, *Chlorella vulgaris*, *Botryococcus braunii*, *Ankistrodesmus falcatus*, and *Tetradesmus obliquus* was compared after their exposure to phosphorus starvation. *C. vulgaris* showed the best performance in terms of biomass growth and nutrient uptake after exposure to P-starvation. Its biomass growth rate increased by nearly 50% after 3-day P-starvation. Its PO_4_ removal rate increased after 3-day P-starvation, reaching > 99% within 7 days. *B. braunii* showed promising performance as it maintained good biomass growth rate and nutrient removal regardless of the P-starvation. Lower performance was observed for other species. 

The estimated biomass phosphate consumption for P-starved biomass showed no change or even a decrease when compared to the reference biomass. This indicates that biomass P-starvation was hindered and did not take the anticipated effect on phosphorus removal rate. 

An enhanced phosphorus uptake was hampered by low N/P ratio, which created nitrogen-limited conditions and made nitrate the preferred nutrient for biomass consumption. Thus, there is a need of strict control over N/P ratio if algae-based wastewater post-treatment is supplemented with P-starvation. In addition, the previously accumulated polyphosphate ensured biomass survival during P-starvation, and it is another factor behind weak P-deficiency condition. The biomass phosphorus starvation did not affect the nitrate removal.

All species showed more rapid alkaline phosphatase activity development at the biomass exponential growth phase, compared to the reference conditions. The AP activity increase is relatable to the decline of PO_4_ concentration and biomass poly-P content. AP activity can be a good indicator to detect and quantify P-deficiency in algae-based wastewater treatment system supplemented with P-starvation for enhanced phosphorus removal.

The results from the present study show that biomass P-starvation can supplement algae-based wastewater post-treatment for rapid phosphate removal. However, its efficiency relies (not limited to) on strict control over physical and chemical parameters (e.g., pH, N/P molar ratio) in wastewater and accurate monitoring of biochemical indicators (e.g., cellular P content and enzymatic activity). 

## Figures and Tables

**Table 1:** Characteristics of wastewater used for the experiment.

Parameter	Unit	Value
Total nitrogen	mg N L^−1^	24.5
NH_4_	mg N L^−1^	0.5
NO_2 + 3_	mg N L^−1^	21.3
Total phosphorus	mg P L^−1^	20.1
PO_4_	mg P L^−1^	17.5
pH		8.2
EC	μS/cm	1600
BOD	mg O_2_ L^−1^	5.3
COD	mg L^−1^	74

**Table 2:** Calibration curves: optical density (OD_680_) to biomass dry weight (g L^−1^).

Species	Equation	R^2^
*T. obliquus*	y = 0.5817x − 0.0129	0.997
*C. vulgaris*	y = 0.4076x – 0.0052	0.999
*B. braunii*	y = 0.5183x – 0.0054	0.989
*A. falcatus*	y = 0.5421x – 0.003	0.992

**Table 3:** Nutrient removal rates and biomass uptake kinetics (mean values, *n* = 3).

	Starvation Period (Days)	Nutrient Removal (%)		Biomass Nutrient Consumption (V), (mg N(P) g^−1^ DW)		Nutrient Uptake Rate (k), (d^−1^)	
		NO_3_-N	PO_4_-P	NO_3_-N	PO_4_-P	NO_3_-N	PO_4_-P
*C. vulgaris*	0	97.7	96.6	57.70	31.70	209.07	114.87
	3	98.9	99.2	29.75	21.12	72.04	51.14
	5	98.1	99.4	34.76	22.12	96.68	61.53
*B. braunii*	0	99.3	97.3	29.19	15.63	72.77	38.96
	3	99.0	99.1	28.17	16.79	66.26	39.50
	5	98.6	99.2	29.23	15.06	68.72	35.40
*A. falcatus*	0	98.7	97.7	44.32	25.95	129.40	75.77
	3	98.4	99.2	35.31	23.88	94.74	64.08
	5	98.7	91.2	49.87	17.89	165.47	59.37
*T.obliquus*	0	10.2	44.5	32.20	111.56	563.02	1.10
	3	10.8	62.4	12.50	49.29	65.66	2.02
	5	3.6	37.8	2.97	5.67	12.65	0.36

## Data Availability

The data presented in this study are available on request from the corresponding author. The data are not publicly available due to privacy reasons.
